# 

**Published:** 1905-04-08

**Authors:** 


### NOTICE TO CORRESPONDENTS.

All MSS., Letters, Books for Review, and other matters intended for the Editor should be addressed The Editor, "The Hospital," The Hospital Buildings, 28 & 29 Southampton Street, Strand, W.C.

The Editor cannot undertake to return rejected MSS., even when accompanied by stamped directed envelope.

**Notice to Advertisers and Subscribers.**

All Advertisements, Orders for Copies of The Hospital, and Business Communications should be addressed to **THE MANAGER (Not to the Editor),** The Hospital, 28 & 29 Southampton Street, Strand, London, W.C.

The Cost of Subscription, post free, is at follows:—Per week, 3½d.; per quarter, 3s. 3d.; per half-year, 6s. 6d.; per annum, 13s. Foreign and Colonial:—Per annum, 19s. 6d., payable, in all cases, in advance.

**NOTICE.—Vol. XXXVII. of "The Hospital" October 1904 to March 1905), in handsome cloth binding, will shortly be on sale at the office of this Journal, price 10s. 6d. Cases for binding the Numbers contained in this Volume 2s. Index to Vol. XXXVII., 3½d., post free.**

**The Monthly Part for April is now ready. Price 1s., by post, 1s. 3d.**

### Medical Appointments.

**William Thomas Briscoe**, M.D., M.Ch.Dub., reappointed Medical Officer of Health by the Chippenham Rural District Council. **J. J. Culmer**, M.R.C.S.Eng., L.R.C.P.Lond., Resident Surgeon Superintendent of the New Providence Asylum, Nassau, Bahamas. **T. V. Cunliffe**, M.D., B.S.Lond., Honorary Pathologist to the Oldham Infirmary. **O. C. M. Davis**, B.Sc., Lecturer on Materia Medica and Practical Pharmacy at University College, Bristol. **W. Sim Garden**, M.B., Ch.B., Second Medical Officer of the County Asylum, Chester. **Alfred Grace**, L.S.A., Medical Officer of the Workhouse and of the First District of the Chipping Sodbury Poor-law Union. **G Hamilton Grills**, B.Ch., M.D., Senior Medical Officer of the Chester County Asylum, Chester. **Robert Thornton Meadows**, M.D., C.M.Edin., D.P.H.Eng. Medical Officer of Health to the St. Germans (Cornwall) Rural District Council. **Newman Neild**, M.B., B.Ch. Vict., L.R.C.P Lond., M.R.C.S., Lecturer on Pharmacology and Therapeutics at University College, Bristol. **Douglas A Reid**, M.D.Edin., M.R.C.S., reappointed Medical Officer of Health of the Borough of Tenby. **Cecil Rowntree**, M.B., B.S.Lond., F.R.C.S.Eng., Assistant in the Cancer Research Department of the Middlesex Hospital. **Hugh Clifford Sharp**, M.B., B.C.Cantab., Deputy Medical Officer of the Truro Port Sanitary Authority. **Thomas Hewlett Worger**, M.R.C.S., L.S.A., reappointed Medical Officer of Health by the Radstock (Somerset) Urban District Council.

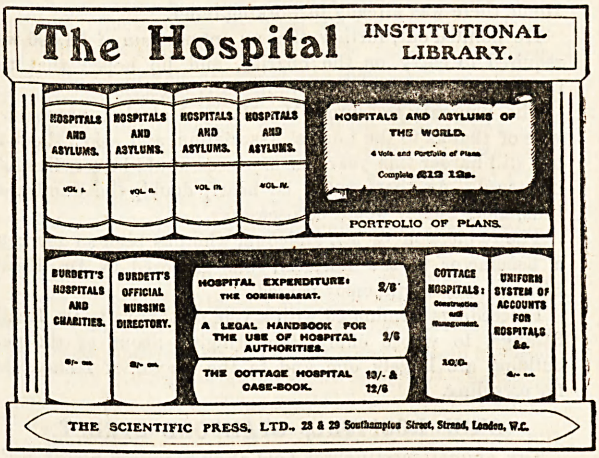

ORIGINAL BATH CHAIRS AND INVALID FURNITURE

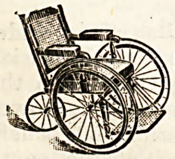

ALL KINDS FOR SALE or HIRE

*Special Discounts. Lists Free.*

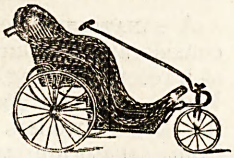

W. MONK & CO., Original Bath Chair Factory, 3, 4 & 5 Broad Street, BATH.

Makers to her late Majesty. Established 50 years.

For GASTRIC ULCER, DYSPEPSIA, MARASMUS.

MOSELEY'S FOOD

6d., 1/-, 2/6.

Samples and Analysis Sent on Application.

FOODS LTD., Dept.F., Stockport.

### A Newspaper for Every Nurse

### A NEW DEPARTURE.

### A NURSING PAPER AT A PENNY.

In response to the repeated representations that have been received from nurses, it has been decided to publish the Nursing Section of "The Hospital" from this date separately at

### ONE PENNY WEEKLY.

This separate Issue will be entitled

### "THE NURSING MIRROR,"

and will, for the present at any rate, contain the same literary matter, advertisements, situations vacant, wanted, &c., as "The Hospital" Nursing Section, which **WILL STILL BE ISSUED WITH** "The Hospital."

### "THE NURSING MIRROR"

will be published each week as a separate paper, and will comprehend all those advantages which long establishment and intimate relations with the nursing world can alone command.

### "THE NURSING MIRROR"

will be published on Friday, and will be obtainable at this Office, and from all Booksellers and Newsagents on that day.

"The Nursing Mirror," "The Hospital" Building, 28 & 29 Southampton Street, Strand, London, W.C.

### For District and Midwifery Nurses.

| | | Post free. |
|---|---|---|
| **HOW TO BECOME A CERTIFIED MIDWIFE.** By E. L. C. Appel, M.B., B.S., B.Sc. Lond. | 1/- net | 1/1 |
| **PRELIMINARY LECTURES ON MIDWIFERY.** By Margaret M. Caley, late Matron, Fulham Midwifery Training School. | 1/6 net | 1/8 |
| **DISTRICT MIDWIFERY CASE-BOOK.** Compiled by Margaret Caley, late Matron, Fulham Midwifery Training School. Suitable size for the Pocket | 6d. net. | 7d. |
| **THE MIDWIVES' POCKET-BOOK.** By Honnor Morten | 1/- | |
| **ON PREPARATION FOR OPERATION IN PRIVATE HOUSES.** By Stanmore Bishop, F.R.C.S. | 6d. | |
| **PRACTICAL HINTS ON DISTRICT NURSING.** By Miss Amy Hughes | 1/- | |
| **THE "SIMPLEX" DISTRICT NURSES' CASE-BOOK.** Containing space for 50 cases, ruled and printed, bound in cloth boards, | 6d. net | 7d. |

| | | Post free. |
|---|---|---|
| **A COMPLETE HANDBOOK OF MIDWIFERY.** By J. K. Watson, M.D. Edin., Author of "A Handbook for Nurses." Profusely illustrated with 143 Diagrams | 6/- net | 6/4 |
| **A PRACTICAL HANDBOOK OF MIDWIFERY.** By Francis W. Haultain, M.D. Profusely illustrated, Tables, &c. Handsomely bound in leather | 6/- net | 6/3 |
| **NURSING NOTES ON MIDWIFERY AND GYNÆCOLOGY.** By Sister Ross, Rotunda Hospital, Dublin | 2/- net | 2/1 |
| **ART OF FEEDING THE INVALID.** By a Medical Practitioner and a Lady Professor of Cookery. With Special Chapter on Diet for Consumptives | | 1/6 |
| **THE POCKET CASE BOOK FOR DISTRICT AND PRIVATE NURSES.** By a Physician | | 1/- |
| **POCKET-BOOK OF TEMPERATURE CHARTS.** Containing 17 Twelve-hour and 8 Four-hour Charts, perforated so that each Chart may be removed after use | 6d. net | 7d. |

**TEMPERATURE CHARTS, MORNING AND EVENING, and FOUR-HOUR CHARTS.** Carriage paid.

| Size 10 inches by 8 inches | | | | |
|---|---|---|---|---|
| | 25 | 1/- | 250 | 6/6 |
| | 50 | 1/6 | 500 | 12/- |
| | 100 | 2/9 | 1,000 | 23/- |

THE SCIENTIFIC PRESS, LTD., 28 & 29 Southampton Street, Strand, London, W.C.

